# "You can save time if…"—A qualitative study on internal factors slowing down clinical trials in Sub-Saharan Africa

**DOI:** 10.1371/journal.pone.0173796

**Published:** 2017-03-16

**Authors:** Nerina Vischer, Constanze Pfeiffer, Manuela Limacher, Christian Burri

**Affiliations:** 1 Swiss Tropical and Public Health Institute, Department of Medicines Research, Basel, Switzerland; 2 University of Basel, Basel, Switzerland; 3 Swiss Tropical and Public Health Institute, Department of Epidemiology and Public Health, Basel, Switzerland; George Washington University School of Medicine and Health Sciences, UNITED STATES

## Abstract

**Background:**

The costs, complexity, legal requirements and number of amendments associated with clinical trials are rising constantly, which negatively affects the efficient conduct of trials. In Sub-Saharan Africa, this situation is exacerbated by capacity and funding limitations, which further increase the workload of clinical trialists. At the same time, trials are critically important for improving public health in these settings. The aim of this study was to identify the internal factors that slow down clinical trials in Sub-Saharan Africa. Here, factors are limited to those that exclusively relate to clinical trial teams and sponsors. These factors may be influenced independently of external conditions and may significantly increase trial efficiency if addressed by the respective teams.

**Methods:**

We conducted sixty key informant interviews with clinical trial staff working in different positions in two clinical research centres in Kenya, Ghana, Burkina Faso and Senegal. The study covered English- and French-speaking, and Eastern and Western parts of Sub-Saharan Africa. We performed thematic analysis of the interview transcripts.

**Results:**

We found various internal factors associated with slowing down clinical trials; these were summarised into two broad themes, “planning” and “site organisation”. These themes were consistently mentioned across positions and countries. “Planning” factors related to budget feasibility, clear project ideas, realistic deadlines, understanding of trial processes, adaptation to the local context and involvement of site staff in planning. “Site organisation” factors covered staff turnover, employment conditions, career paths, workload, delegation and management.

**Conclusions:**

We found that internal factors slowing down clinical trials are of high importance to trial staff. Our data suggest that adequate and coherent planning, careful assessment of the setting, clear task allocation and management capacity strengthening may help to overcome the identified internal factors and allow clinical trials to proceed more efficiently.

## Introduction

Clinical trials are essential for medical advances as they provide the highest degree of evidence to support new interventions and decisions about disease management. However, the conduct of clinical trials is very complex; people are exposed to potential health risks and vast quantities of data are collected. Professionals working in clinical trials are confronted with numerous regulations, ethical challenges, high workloads and administrative requirements. Over the years, the costs, complexity, legal requirements and documentation associated with clinical trials globally has risen constantly [1–5], however, the added value of these changes in terms of increasing the quality of clinical trials remains unknown [6]. This trend stands in sharp contrast to current efforts to make health systems more productive.

Working conditions are even more complex for clinical trials conducted in resource-limited settings. This paper focuses on Sub-Saharan Africa (SSA), where limited infrastructure, human resources, experience and ethical challenges may especially affect the efficient conduct of clinical research. The topic of efficient trial execution is of particular importance in these settings as the number of clinical trials carried out in SSA is rising [7, 8] while funding and the number of qualified health staff remain limited. In 2014, the total annual funds available for neglected disease medicines development was USD 3,377 million in [9]; in the same year, the estimated cost to the pharmaceutical industry of developing one new prescription medicine to the point of marketing approval was USD 2,558 million [10]. Increasing efficiency in trials would allow more trials to be conducted with the limited funds available. This in turn has important implications for public health in resource-limited settings, where trials are urgently needed to develop new safe and effective health interventions [11].

Increased efficiency in clinical trials would not only reduce costs, but also lead to more productive work settings with manageable workloads and requiring less time to perform a trial. The International Conference of Harmonization`s Good Clinical Practice (GCP) Guideline E6, the most widely accepted standard for the conduct of clinical trials, has been amended in 2016 for the first time since its introduction in 1996. The main reason for the addendum in preparation is “the encouragement of implementation of more efficient approaches to clinical trial design, conduct, oversight, recording and reporting” [12]. By increasing efficiency for clinical trials in SSA, we do not mean to lower the standard for these trials, ethical soundness is essential, but to identify and manage factors which are currently slowing down trials.

There is little scientific evidence to show that the procedures for clinical trials are carried out in an efficient and cost-effective way [13]. The few publications addressing the conduct of clinical trials in resource-limited countries are mostly reflections on past trials. These publications are not directly reporting on factors slowing down clinical trials, but list general challenges. A particular challenge reportedly associated with clinical trial delays is the lengthy regulatory and ethical review process [2, 14–16]. The complexity of the latter is compounded by multiple ethical reviews and communication gaps between the committees and authorities [14, 17]. Promisingly there are developments towards a better collaboration and joint reviews between these bodies; the WHO-supported AVAREF (African Vaccine Regulatory Forum) platform was founded to support multi-national vaccine trials, but also was instrumental in the acceleration of clinical trials during the Ebola crisis [18]. Other reported challenges include the often poor and/or illiterate study participants and differing cultural values and beliefs [8, 19], which raise ethical questions [8, 20] and may lead to recruitment, consent and follow up difficulties, which slow down trial progress [8, 17, 21]. Inadequate infrastructure, particularly in rural areas, may affect clinical work, communication, access and the availability of basic refrigerated medicines, which together may also considerably slow down trials [21, 22]. The aforementioned challenges can be categorised as external factors, as they are the given conditions of the framework in which clinical trials operate in these settings.

In this manuscript, we focus on those factors that slow down trial progress and that exclusively relate to clinical trial teams and sponsors, defined here as internal factors. Such factors may be influenced independently of the external conditions, if the challenges are known and the parties are aware of them. Only a few published reflection papers mention such internal factors affecting clinical trials in SSA and most of the factors described were of general importance and not particularly targeted to the time component of trials. For example, brain drain and inadequate budgets were described as internal challenges in vaccine trials in Africa [8]. The authors noted that investigators were often frustrated about their small scientific output and recommended more cooperation among stakeholders. Experiences from the Gambia pneumococcal vaccine trial demonstrated that human resource management was very time consuming [23]. The same authors identify lessons learned, citing the importance of a quality management plan, of documenting roles and responsibilities of collaborating groups and of on-site supervision including feedback to each staff member. They also stressed the need for senior staff to create a strong team spirit. Adequate planning, including assessments of available resources, was considered indispensable. To the best of our knowledge, apart from these reflection papers, there is only one research study on this topic. A qualitative study focusing uniquely on investigator-initiated trials in Ethiopia identified internal challenges such as limited learning opportunities (which negatively affects human resources), lack of recognition and career options, lack of experience, poor planning and problems with trial management [16]. In other reflection papers, high administrative requirements resulting from a conservative interpretation of guidelines and regulations were blamed for increased duration and costs of trials [2, 24]. In contrast, own research found that clinical trial teams in SSA do not perceive the administrative requirements as slowing down the trials but rather considered them essential for ensuring quality [25].

A lack of data on the operational aspect of trials was stated in the literature [23, 26] and, to our knowledge, no publication has specifically investigated how efficiency could be increased internally in trials. By identifying internal factors that slow down clinical trial progress, we take a first step towards increasing efficiency and achieving more resource-effective trials. Compared to external factors, internal ones may be easier to influence, manage or eliminate. Trial teams have an important role in overall trial success and are faced with complex trial processes on a daily basis. Hence, they were considered an important source from which to gain valuable insights into the challenges to and opportunities for increasing the efficiency of trials. The aim of this study was to investigate internal factors slowing down clinical trials by giving a voice to trial teams in SSA.

## Methods

### Study setting

Qualitative data were collected in clinical research centres in Kenya, Ghana, Burkina Faso and Senegal. These four countries were selected in order to compare results between different language and geographical regions. All four countries strongly contribute to health research in Africa. We contacted all major clinical research centres specialising in poverty-related diseases and with a track record of completed clinical trials (no more than four such centres could be identified per country). In each country, we conducted our study in the first two research centres to agree to our visit. We visited both rural and urban clinical research centres. Seven centres were dedicated research institutions and one was a hospital`s department performing clinical research. Visited centres mostly conducted externally sponsored research, however, six out of eight centres has conducted investigator-initiated trials, too. The names and detailed locations of the centres have been purposely withheld to allow participants to remain anonymous.

### Sampling

**Fig 1:**
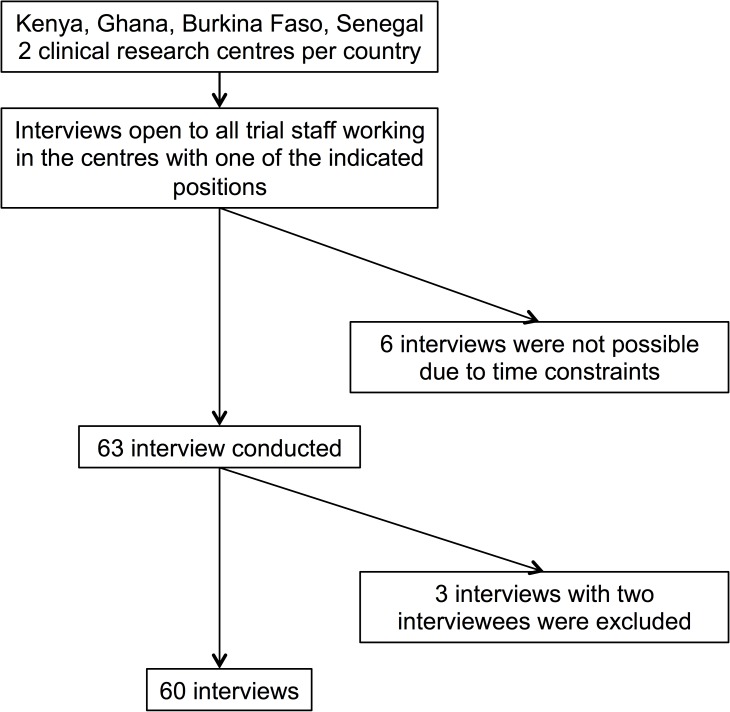
Sampling procedure.

At the centres, interviews were open to all investigators, study coordinators, clinicians and quality assurance professionals with at least six months of experience in clinical research. The different organisational levels were selected to enable data triangulation. For each centre, the sample was purposefully drawn with the assistance of a senior clinical trial staff member, who acted as gatekeeper by approaching eligible participants and informing them about the study. Nobody refused participation but six trial workers were unavailable for interview due to time constraints during our visit (see Fig 1). In one centre, six participants asked to be interviewed in groups of two. These interviews were conducted, transcribed verbatim and the findings were in line with the overall result but ultimately the data were excluded to avoid a possible bias. Interviews lasted on average for about 45–60 minutes.

### Interviews

A semi-structured interview guide (see S1 Text) with open-ended questions was used to encourage participants to describe their own understandings and opinions and to allow identification and exploration of themes and hypotheses that might not have been anticipated. The interview guide was developed by an interdisciplinary team based on a literature review and preliminary interviews with clinical research professionals. The interview guide was pre-tested outside the study area and later developed iteratively as data emerged. The guide was used with flexibility and included general questions about quality, challenges and perceived inefficiencies in clinical trials. Interview questions did not target experiences in a specific trial but rather the participant`s trial experience in general and excluded trials involving contract research organisations (CROs). In Kenya and Ghana, interviews were conducted in English. After translating the guide into French (including back-translation and revision of terminologies), interviews were conducted in French in Burkina Faso and Senegal. Translations were carefully discussed with all team members and local partners in order to use locally adapted terminologies and to avoid misunderstandings. Data were collected by NV alone in Ghana, together with ML in Kenya and together with AJ in Burkina Faso and Senegal, between April 2014 and September 2015. NV was a pharmacist and PhD-candidate while ML and AJ were master students. All data collectors were female and Swiss. Interview-partners were informed that the interviews were part of a PhD-project. There was no need for a translator as data collectors spoke English and French. The interviews took place in a private room of the clinical research centre allowing for confidentiality. Summaries and observations were written down in a field diary by the interviewer directly after each interview. These notes facilitated self-reflection and were useful during data analysis. Saturation of information was reached when few or no new concepts were raised [27].

### Data management and analysis

All interviews were transcribed verbatim. NV reviewed the English and AJ the French transcripts and original recordings. Data were grouped according to the interviewees’ responsibilities and countries. Thematic analysis was conducted as per Braun and Clarke 2006 [28] using MAXQDA 11 software. English and French transcripts were analysed in their original language. After repeated readings of the transcripts, the first and the third author performed initial coding by coding important features of the gathered data in a systematic matter and amassing data relevant to each code. The first author had the lead during data analysis. The coding framework was discussed amongst co-authors (CP is a social scientist) before agreeing on a final version. The analysis focused on internal factors perceived as slowing down clinical trials. Notes were taken during the analysis to ensure that analysis was reflective. Themes and sub-themes that emerged from the subsequent data interrogation were tested in further interview analyses. Similarities, differences and patterns were identified across the interviews and sub-themes which gave structure to the over-arching themes were refined. The data set was re-read to check for coherence of data within themes and for clear and identifiable distinctions between themes and sub-themes. Finally we went through the data set to code any additional data that had been missed in previous coding stages. After discussions with experts from various disciplines we decided on final definition and naming of the themes and sub-themes.

### Ethical considerations

Ethical review exemption was obtained from the Ethics Committee of North-western and Central Switzerland (EKNZ) and from the Pharmacy and Poisons Board in Kenya (Ref. No. PPB/ECCT/Misc/2015(79)), based on the reasoning that the research project did not involve access to or collect private, sensitive or health-related data or materials. The ethics committees in Ghana, Burkina Faso and Senegal were asked to grant an ethical exemption but their statutes do not allow for such exemptions. Therefore, we applied for and received full ethical clearance from the Ghana Health Service Ethical Review Committee (GHS-ERC: 18/09/14), the Comité d`Éthique sur la Recherche en Santé in Burkina Faso (N 2014-11-131) and the Comité National d`Éthique pour la Recherche en Santé in Senegal (n12/MSAS/DPRS/CNERS).

An information sheet about the study was given to the participants prior to the interview. We explained the objective and background of the research project and informed them of their right to leave the study any time. Anonymity and confidentiality were guaranteed, thus no study centres and names are disclosed in the paper. Written consent was obtained in Ghana, Burkina Faso and Senegal. In Kenya, audio recorded oral consent was sufficient as the study received ethical review exemption. A copy of the consent form, including contact details of the interviewer, was given to the participants. All participants agreed to be audio recorded during the interview, which averaged 45 minutes.

This study adhered to consolidated criteria for reporting qualitative research (COREQ) [29].

## Results

### Study participants

A total of 60 clinical trial staff participated in the key informant interviews. Thirteen to seventeen interviews were conducted in each country (see Table 1). Saturation of information was reached as little or no new concepts were raised after the first 11 interviews in each country.

**Table 1:** Characteristics of participants.

		Kenya (n = 17)	Ghana (n = 13)	Burkina Faso (n = 16)	Senegal (n = 14)
**Role in trial**	Investigators(n = 28)	8	4	8	8
	Study coordinators (n = 17)	5	6	3	3
	Clinicians(n = 10)	3	2	3	2
	QA professionals(n = 5)	1	1	2	1
**Gender**	Female (n = 20)	9	4	3	4
	Male (n = 40)	8	9	13	10

Most participants had been involved in commercial and non-commercial drug and vaccine trials (see Table 2). Participants had between 10 months and 15 years of working experience in clinical research.

**Table 2:** Participants` experience of working in clinical trials.

		Kenya	Ghana	Burkina Faso	Senegal
**Clinical research experience**	0 to 2 years	1	4	2	1
	3 to 5 years	2	3	4	2
	6 to 8 years	6	0	5	3
	9 or more years	8	6	5	8
**Study phase**	Phase I (a or b)	10	3	10	3
	Phase II	12	3	13	4
	Phase III	13	10	13	8
	Phase IV	4	7	9	3
**Type of trial**	Drug trial	15	8	16	11
	Vaccine trial	14	10	13	9

### Themes

We identified several internal factors that are perceived to slow down clinical trials. The two overarching themes were (1) planning and (2) site organisation, which are described below with subthemes.

### Planning

Clinical trial staff, independent of country and position, repeatedly stressed the importance of the planning phase for clinical trials. More than half of all respondents (41/60) stated that in order to be more efficient, enough time should be allowed for planning. In their experience, things that were not thought through in the planning phase led to lost time during the trial. Staff frequently mentioned that they lose time in their trials due to poor planning.

“Sometimes so much does not go into planning. If you really would spend so much time in planning, it would be a lot easier. So if you plan a lot regarding processes, procedures in the study then you wouldn’t have to rush or you wouldn’t have to make some mistakes because you have duly planned, so it is sometimes a bit challenging when you've not really planned so well. Then that is where much of the problem is.”

—Study coordinator, male, Ghana, centre two

Various planning sub-themes were frequently discussed such as budget feasibility, clear project ideas, realistic deadlines, understanding of trial processes, context adaptation and involvement of site staff in planning. They were mentioned to the same degree across the different professional levels interviewed.

#### Budget feasibility

Seventeen participants, mainly from the French-speaking countries, mentioned that budgets were not carefully developed in the planning phase and were based on unrealistic or incorrect assumptions. Interviewees said that trials in Africa are expensive due to additional associated costs like community engagement, electricity and the trial participants’ health care. Under-budgeting was perceived to slow down trials by either stopping the trial completely or by distracting site staff during the trial with budget discussions or with applications for new funding sources. According to the interviewees, diligent elaboration and thorough discussion of the budgets in the planning phase would help to save time once the trial is in process.

“Often people are mistaken in the estimate, because they have not budgeted these things, because they have not thought about this before. And often Europeans say, yes it's expensive, you ask too many things. No, because the environment is different, this has to be considered.“

—Study coordinator, male, Senegal, centre one

#### Clear project idea

Five respondents from French-speaking Africa mentioned to lose time because of changes to the project by request of the sponsor during the project process or implementation phase. One Burkinabe clinician asserted that sponsors should develop a clear view of the project goals and approaches in partnership with the sites. Such an approach would avoid time losses due to amending the project according to the sponsors’ new ideas during the production phase.

#### Realistic deadlines

In nine interviews, participants suggested that unrealistic deadlines defined in the planning phase slowed down clinical trials. Trial staff mentioned that sponsors push to have the first trial participant enrolled as early as possible, although the deadline might be unrealistic. Starting a trial without being fully prepared increased the probability of making mistakes during the trial conduct. Such mistakes required adjustments to be made and were time consuming to resolve. Common pitfalls leading to misestimating timelines were ignoring the long approval process and being too optimistic when calculating recruitment rates (a phenomenon commonly known as Lasagna’s Law). Deadlines of great importance to the sponsors were often unrealistic to achieve and forced trial teams to rush during preparation, creating extra burdens.

“So it is that at some point you may, for example, start when you are not sure that everyone is ready. But you must start with the team and then train people in the study. This only lengthens the time of the study.“

—Investigator, male, Burkina Faso, centre one

According to interviewees, if the deadlines were realistic, the long waiting time before approval is granted could be used more efficiently. A study coordinator described the centre’s positive experience of using this long waiting time for diligent trial preparation. According to her, without making use of the waiting time before approval, the site would have experienced all sorts of problems later on. On a different level, two investigators complained that the negotiation processes to establish the contract between the trial site and the sponsor frequently delayed the trial start.

#### Understanding of trial processes

Providing sufficient time for every team member to understand the protocol, as well as their role and responsibility in the trial, was perceived to be crucial. The idea came up mainly in Kenya and Senegal (17/60). The importance of joint team meetings to go through every step in detail and anticipate possible challenges of the trial was also stressed. Interviewees reported that elements that had not been anticipated and pre-discussed in the preparatory phase would slow the trial down later. Five staff members shared with us the benefits they experienced from conducting a test run with a dummy participant to practice all trial steps and identify hiccups prior to the start of the study.

“I think we do not put people enough in the situation of a real life trial before starting a clinical trial. We think that because we are doctors, we will know how to do that. It is not that simple."

—Study coordinator, female, Senegal, centre two

#### Context adaptation

Adapting the project to the setting was reported to be the single most important consideration that was often neglected during planning. The importance of context adaptation or the challenges of unadapted trials came up frequently (28/60) across different staff levels and countries, but was raised somewhat more frequently in the French-speaking countries. Staff defined adaptation as ‘adapting the project to the trial participants’ culture and values as well as to the health system, site specific procedures, seasonal conditions and the human and infrastructural resources available’. Respondents suggested checking, for example, if the equipment was available for certain lab tests or ensuring that the trial was as uninvasive as possible due to trial participants’ fear of having blood drawn. According to interviewees, unadapted studies were the result of Northern sponsors`unfamiliarity with the local context. A frequent claim was that sponsors still assumed that all of Africa was the same and were not aware of the different realities in different countries or regions. This misperception resulted in great efforts during implementation to correct for, efforts which could have been saved if the project had been adapted to the setting from the outset (during the planning phase). In one respondent`s experience, protocols that are both unadapted and stringent were very difficult to implement and often resulted in multiple protocol deviations.

“I would tell you to try to really adapt to the realities of the countries. Because if you give us a typical European protocol that has to be reproduced here, I think we are going to have problems. We do not have the same manner of working. We do not have the same tools to work with. So it might be important to really see what is feasible in the country (…) If not, you will have many, many deviations afterwards, because we were not able to do that. We would need all the time to document why we were not able to do that because we did not have the lab to do this test or that test.”

—Study coordinator, female, Burkina Faso, centre five

“It is challenging working with people [when] they don’t have experience with this type of setting”

—Investigator, female, Kenya, centre two

Interviewees appreciated feedback meetings with sponsors to discuss challenges of previous trials to avoid repeating mistakes in upcoming trials. A Ghanaian clinician highlighted her positive experience of adapting the protocol to the site in a multicentre trial, a difficult task as all sites work along the same protocol, despite differing contexts.

“If there is a protocol for about seven different African countries to run a trial in every country it’s reviewed in (…) the scientific review committee (…) it’s also adapted to how we run our system, some parts are adapted. So what I know is that, in all the various countries we have one parent protocol but in the protocol we are allowed to make adaptations to suit our health system, then it becomes a workable protocol.“

—Clinician, female, Ghana, centre one

#### Involvement of site staff in planning

Involving local clinical trial staff in the planning process was, for many respondents (35/60), the best way to ensure that the trial is adapted to the local situation as local staff are most familiar with the context. The topic was raised across all staff levels but with higher frequency in the French-speaking countries.

One Burkinabe clinician stated that all the time spent adapting the trial to local practices and conditions could be saved if the trial staff were involved in the planning phase. Interviewees mentioned that local trial staff would help to identify unadapted processes as well as risks, difficulties and redundancies. Respondents suggested developing local risk management plans. For many interviewees, involving site staff in the planning also means involvement in protocol development. More details regarding site staff`s involvement in protocol development will be reported elsewhere (Vischer *et al*., forthcoming). However, the notion as discussed here included planning and implementation in general.

“There are studies (…) that people have designed together, people were involved in writing, it makes that this loss of time on the field is not felt. But when it is, like for example firms, I will not mention names, that send their protocol and say ' this is what we want, this is the information we want you to collect ', this is when the losses of time happen.“

—Clinician, male, Burkina Faso, centre two

In contrast to the statements above, six interviewees were satisfied with their degree of involvement in the trials`planning. They argued against more involvement as they perceived that it would be impossible to foresee every risk, even for trial staff, and that the involvement would add too much work. Three of the six interviewees were from the French-speaking countries and held high ranking positions. However, there was no consensus within the centres as other interviewees from the same centres complained about the lack of involvement.

### Site organisation

The theme of site organisation incorporates topics such as staff turnover, employment conditions, career path, workload, delegation and management. All of these topics were mentioned as factors slowing down clinical trials.

#### Staff turnover

In 19 interviews, high staff turnover was mentioned as a major internal challenge contributing to losses of quality and time. All but two clinical research centres (in Senegal) perceived staff turnover as a limitation. Interviewees reported that former staff left for other clinical research centres, contract research organisation and positions abroad. Only one interviewee regarded circulation of staff as positive and healthy and said that employers had to accept that they train staff for others. The majority (33/60) stressed repeatedly the importance of experienced and qualified staff for guaranteeing good quality, avoiding mistakes and inefficiencies as well as for supervising inexperienced staff, indicating the negative influence of losing experienced staff. Finding qualified and experienced staff is challenging due to the lack of health professionals in SSA, according to respondents. As the conduct of clinical trials is not part of basic health professional training, great efforts are made to train new staff in research concepts and to prepare them for the strict working environment.

"In our daily practice, what makes us waste time is especially the repetition of staff training."

—Investigator, male, Burkina Faso, centre one

“So when you have very experienced people leaving, that can cause a very great challenge.”

—Investigator, male, Kenya, centre two

#### Employment conditions

One stated reason for high staff turnover was that staff was hired temporarily and if there was no subsequent trial in the centre, the employee had to leave to find new employment.

“You recruit people, you train them for temporary employment (…) The drug trial, maybe it will not exceed eight months. After eight months, you are not sure if you keep the person (…) He goes somewhere else or he will look for something. You're going to work on another project; you will find other people who perhaps will be taken away elsewhere. And there is staff turnover. We, as such, we are in the institution, we are working for the institution, there is no problem. But the support staff is renewed all the time. And that doesn’t help. If we had permanent staff, I think, with time they will acquire some experience and it will also allow them… there will be mistakes they won’t make anymore.“

—Investigator, male, Burkina Faso, centre one

A few interviewees expressed dissatisfaction working in clinical trials because of the many routine tasks, the very strict working environment and the need to work under high pressure. The resulting low staff motivation prevented efficient trial conduct.

#### Career path

High staff turnover was also attributed to lack of recognition and career prospects in clinical research. A Kenyan investigator said that after having worked in a position for a while, you want a promotion, but instead you stay in the same position for many years. The need for a career path for clinical trial professionals came up in six interviews. Poor career prospects were seen as contributing to staff leaving for salary reasons as soon as they had gained enough experience. The better-paid jobs were often outside of SSA, contributing to brain drain. This was only mentioned when the interviewers actively asked participants in a follow-up question to give reasons for high staff turnover rates.

#### Workload

Related to site organisation, interviewees perceived high workloads as another major factor slowing down clinical trials. With a lot of emotion, respondents (23/60) reported that the high workload was an enormous challenge for them. Overloaded staff sadly reported that they had lost their social life, had not had holidays for five years, or were involved in eight studies at the same time, for example.

“That is to say, you can work 24 hours 7 days a week without even having time to eat or sleep. It is difficult, but I am used to it today. I’m used to it. Even at 2 am you wake me up, I'll do what is required.“

—Investigator, female, Burkina Faso, centre one

Independent of their position, we interviewed overloaded and stressed staff members in each country. We identified them by their statements or by their difficulty of finding time for an interview while we were there. In contrast to the interviewees that were constantly overworked, other interviewees were not overloaded or less so, particularly in centres without on-going trials. A female Senegalese study coordinator summarised this situation as follows: “Sometimes there is a rush and sometimes there is not much going-on in the centre”.

Participants shared ideas for reducing the high workload and associated time losses. Firstly 10 interviewees, mainly from rural research centres, suggested hiring more staff in order to distribute the workload among more staff members. However, they knew that this was challenging due to a lack of qualified personnel and little interest in working in rural areas. Secondly, a few interviewees, mainly from one centre, suggested distributing the trials more evenly throughout the year instead of, for example, only focusing on malaria trials, which all take place during the rainy season. Thirdly, four interviewees suggested setting realistic deadlines to avoid a constant sense of urgency. Lastly, their strongest suggestion for reducing high workloads was to assign clear roles and responsibilities for everybody involved in the trial. They indicated that good trial coordination would enable fair sharing of the workload and ultimately save time. All interviewees shared the opinion that delegation helped to guarantee a manageable workload.

“So that's it. There is the project manager, there is the research assistant and there is the technician of study. The work is divided. There is not a too high workload.”

—Clinician, male, Senegal, centre two

Interviewees mentioned that clinical trial responsibilities are concentrated among investigators in addition to their medical and scientific tasks, which adds work to their already overloaded schedules and consequently leads to delays. Hence, delegating tasks is particularly important to relieve investigators. This observation was reported mainly in the French-speaking countries. Table 3 shows that a study coordinator position (or similar role) hardly exists as a single role in these countries and, thus, investigators often have a double responsibility as study coordinators.

**Table 3:** Additional or parallel roles in trials.

	Kenya	Ghana	Burkina Faso	Senegal
**Investigators (in addition):**study coordinator (n = 4)	-	-	3	1
**Investigators (in parallel):**lab manager (n = 2)	-	-	2	-
**Study coordinators (in parallel):** lab manager (n = 3)	-	-	1	2

“You can save time if for each investigator you put a study technician who helps him. This is not done here.“

—Investigator, male, Senegal, centre two

#### Management

Interviewees reported that clinical research centres required management to avoid challenges associated with slowing down clinical trials. This overarching topic was not reported as frequently (7/60) as the more specific ones like high staff turnover and workload but good management was seen to influence and even prevent the latter ones. For example, the elimination of gaps between trials was considered to be a management task and would reduce staff turnover rates. This issue was particularly raised in centres focusing on seasonal illnesses like malaria. Other important managerial aspects mentioned were negotiating and budgeting skills. Particularly for discussions with the sponsors, such skills were regarded as essential to defend the budget, for example. Good coordination, including good staff coordination and the creation of a team spirit, was considered to be primarily a management task.

A few interviewees suggested having centre managers to facilitate trial conduct in a reasonable timeframe through responsibilities for acquiring new projects, ensuring staffing, maintaining budgets and communicating with the sponsor. One investigator compared her centre with another one as follows:

“*They have a manager and you know that helps a lot*. *And for us*, *I see us going that way because the more you do many multiple studies at the same time you just can’t keep on doing it the way we have been doing it where the PI or the main physician is burdened with all those details*.*”*

—Investigator, female, Kenya, centre two

Another idea for improving institutional management skills was to educate investigators in management.

“So that's all these skills that you need to have, not only clinical expertise, lab competence, but also the competence of management.“

—Investigator, male, Senegal, centre one

The management topic was mainly raised by high ranking staff members working in the French-speaking countries.

## Discussion

To the best of our knowledge, this is the first study to investigate internal factors slowing down clinical trials in SSA. The literature on the topic is scarce and mainly focuses on external challenges like the lengthy approval process, which is often described as a major cause for delays in clinical trials [2, 14]. We identified several internal factors (factors only relating to clinical trial teams and sponsors) that were perceived to slow down clinical trials; we summarised them according to two themes, “planning” and “site organisation”. Based on our results, we argue that trial efficiency could be increased by tackling these internal factors.

It surprised us how clearly and consistently these two themes emerged from the interviews, independent of position and country, and also how often internal factors slowing down clinical trials were mentioned in general. The openness of the qualitative approach allowed for in-depth exploration and enabled respondents not only to list challenges but to elaborate on possible solutions as well. Interviewees presented several solutions which would not have been possible with a closed questioning format. Factors inhibiting efficiency were often associated with a decrease of quality, indicating that improving “planning” and “site organisation” might increase trial quality as well as efficiency.

It was striking to note the frequency with which poor planning came up in interviews as a factor slowing down clinical trials. In drug development, speed is considered imperative because of very high costs and frequent competition. As a result, sponsors and funders pressure teams to meet tight deadlines. However, trial teams see this practice as actually resulting in time losses, which contradicts the general view that applying pressure increases efficiency and may explain the persistent challenge of poor trial planning Our observation is, however, in line with Senge`s Law of Systems Thinking, which states that “faster is slower”. Senge warns against the temptation to advance at full speed without caution, since every system has its own unique and optimal speed and a fast fix often leads to a slow cure [30]. J. Brock-Utne reports on his clinical research experience and highlights that “before you embark on your question you must prepare well, which will take much longer than you think” [31]. The result is further supported by literature stating that intense planning in clinical trials is particularly important in resource-limited settings [17, 26, 32]. The process map available on the global health network webpage shows that planning clinical trials is important and lengthy [33]. The forthcoming three process map steps are in line with our findings: I) the importance of having a clear project idea and one single question; II) the importance of realistic trial costing and secured financial support, additionally this point is supported by other authors who discuss the difficulty of predicting budgets due to unstable currency [8], for example, and who complain about the limited flexibility of sponsors and funders over budget [14, 26]; and lastly III) the importance of meetings of study staff in order to understand and discuss every trial step before the trial. Our interview partners requested very clear instructions and exchange. The GCP-guideline does not specifically require standard operating procedures (SOPs) for investigators, however, SOPs supported by study specific working instructions might mitigate this concern. In addition, we argue that having a checklist for every trial-specific step, once the trial participant is on site, would be useful for staff and ensure uniformity of how tasks are performed. The newer trend of assessing risks in preparation for a clinical trial [34] might be an ideal way to improve planning and to set more realistic timelines in such complex working environments.

Interviewees particularly stressed the importance of adapting the trial to the context during the planning phase. A possible explanation is that trials in SSA have often had Northern sponsors who might not be familiar with or ignore setting differences. The literature confirms trial staff opinions that adapting projects to the context prevents time-consuming errors and challenges along the way [8, 21, 35]. Challenges of unadapted trials were reported more often in the French-speaking countries. This could be the result of increased language barriers, as protocols and communication with sponsors are often in English. Our data suggest that sponsors should thoroughly inform themselves about local contexts, carefully assess the framework and inquire about what went wrong in previous trials. We argue that this would allow sponsors to develop innovative strategies for the respective settings.

Respondents suggested involving the local staff in planning to increase trial suitability. It is a peculiarity of clinical trials that the investigator (i.e. the site) and the sponsor have clearly defined roles [36]. The sponsor’s role is very prominent and limits the influence of the investigator / research site on decisions about the design and conduct of trials. In turn, the sponsor is expected to thoroughly understand the capacities, limitations and requirements of the site to carry out the project. There is evidence in the literature about the advantages of having involved local trials teams in SSA [17, 37]. A recent publication on lessons learned in Ebola trials reports on the importance of having foreign researchers engage with appropriate local stakeholders at the earliest stage possible [38]. Systems thinking stresses that stakeholders will know what problems are most likely to arise and stakeholder should be involved from the beginning [30]. The transboundary research principles of the Commission for Research Partnerships with Developing Countries (KFPE) recommend setting the agenda together with stakeholders and to interact with stakeholders [39]. Particularly local staff’s input on recruiting and following-up trial participants can potentially speed up trials [22] while adaptation to the site’s procedures and routines accelerates the implementation of the trial. We are aware that extensive site staff involvement is not feasible for multicentre trials but we are of the opinion that having at least one staff member per site involved in the planning process is important to account for different settings [26, 40]. It would then be the responsibility of this staff member to seek inputs from the rest of the team.

The second theme to emerge was “site organisation” and included staff turnover, employment conditions, career path, workload, delegation and management. Interviewees were highly affected on a personal level by human resource challenges like high staff turnover and workloads, which might explain the frequent reporting of this topic. Staff turnover is often a challenge where financial incentives [41] and lack of job security [17] are prevalent. Consistent with Angwenyi *et al*. interviewees perceived experienced staff as crucial for the supportive supervision of and as role models for the many inexperienced staff [32], indicating the challenges of losing experienced staff. Staff turnover is generally a challenge in health facilities in resource-limited settings as it is associated with increasing workloads, lowering the quality of services, reducing team efficiency and causing a loss of institutional knowledge [42]. The missing career path of African clinical scientists is mentioned throughout the literature [7, 8, 13–15]. In addition, clinical scientists do not have a high status [13], which discourages professionals from entering this career [7, 14, 16]. Usually, only the principal investigator’s name is visible and recognition of the rest of the trial team is absent [43]. Whitworth *et al*. argue that the lack of career paths to attract and retain good researchers is the most serious impediment to health research in Africa [44].

A few interview partners directly mentioned the importance of management in order to save time in clinical trials. We argue, in turn, that all site organisation factors slowing down clinical trials are influenced by management. The WHO stresses the need for management in the health sector, including management of volume and coverage of services, resources (staff and budgets) and external relations and partners [45]. Accordingly, building a portfolio, preferably going beyond a single disease, is crucial for the sustainability of a trial centre [17]. This is a management task that could decrease staff turnover by guaranteeing permanent positions and a balanced workload. Cutts *et al*. confirmed the importance of management and noted that it takes up a large proportion of time in clinical trials [23]. This opinion is shared by the 2014 report of the European and Developing Countries Clinical Trials Partnership (EDCTP) on capacity development for clinical research in SSA, which recommends to go beyond scientific issues and to address managerial skills [46]. Whitworth *et al*. recommend providing institutional support for management of research centres [44] and the Council on Health Research for Development (COHRED) more specifically recommends improving the contract management capacity of these institutions [47]. Our data suggest that management should focus on winning staff commitment, creating an area of expertise and using human resources optimally by allocating clear tasks to appropriately trained and suitably qualified professionals.

The issue is generalisable and lack of management has been described as a common challenge in health systems in resource-limited countries [45, 48]. At the same time, increasing evidence shows that good management practices can generally improve health system performance by increasing institutional incomes and patient satisfaction levels, among other things [49, 50]. Health professionals, including clinical researchers, are not generally trained in management and we support the implementation of widespread management training to improve both institutional management skills as well as career prospects. To improve management, WHO recommends classroom or online training courses and the inclusion of basic management concepts in the training programmes of nursing and medical schools [45].Effective on-the-job methods for improving management also exist and include learning-by-doing and action-learning through regular supportive supervision of high level managers or twinning between similar organisations in developed and developing countries, for example [45]. Clinical trials and long-term partnerships may offer room for such training opportunities.

There may not always be a very clear distinction between internal and external factors. Some of the described internal aspects may under certain circumstances as well be influenced by factors which are not under control of a study site or sponsor. First, in non-commercial trials deadlines and budget feasibility are mainly influenced by the external funding agency rather than the sponsor [51]. However, by improving the site`s negotiations skills the feasibility of the budget could be improved internally. Second, missing context adaptation does not only need to be addressed internally by sponsors and trial centres but also by Ethics committees and Drug Regulatory Authorities. These institutions have a responsibility that only locally adapted trials get approved. Third, career paths of clinical researchers depend on the funding available in a certain field of research, but clearly also by the centre capability to build a portfolio of trials and the willingness to promote promising staff and to delegate responsibility.

With the exception of quality management plans and documenting responsibilities of collaborating groups, neither of which came up in our interviews, our study confirms all of the factors slowing down clinical trials as mentioned in the few reflection papers on the topic.

We found that that identified challenges of trials in sub-Saharan Africa have also been reported in High Income Countries, such as high staff turnover [52], poor career prospects [53] and a lack of management [40]. McMullen *et al*. explained differences in recruiting performance of sites conducting complex intervention trials in a high-income setting and yielded results similar to ours [54]. They report that centres with good recruitment rates were characterised by strong leadership and by good relations between management and staff and among staff. Support and time for implementation, appropriate division of roles, stable staff, and consideration of site-specific characteristics and realities were deemed crucial.

Our study must be considered in light of a few limitations. We only investigated the perception of trial site staff without comparing it to the sponsor`s perception. The interviewers were female, Swiss scientists. Trial staff might not have been keen on talking about weaknesses in trial conduct with the interviewers as auditing and monitoring visits are often carried out by foreigners too. In order to deal with this, staff members were encouraged prior to the interview to speak openly and anonymity was ensured multiple times. In turn, we gained confidence about the evidence presented as we consistently identified the same two main themes independent of country and staff level. Another limitation was that we selected the first two centres, that agreed to our study while findings from the centres that were more hesitant to agree might differ. Qualitative research is constrained in terms of its generalisability; to mitigate this shortcoming we conducted the study in four countries and two languages of sub-Saharan Africa.

This study is intended to start a debate about efficient processes in clinical trials. We argue that study optimisation and future research should not only consider external but also internal challenges to conducting clinical trials. Particular topics of interests are how to improve the planning process, how to involve clinical trial staff in planning in a feasible way and what are quality criteria in clinical trials conducted in resource-limited settings. Further, we encourage future research to investigate how to make clinical research careers more attractive. We found that the experiences of local trial staff are a valuable source of information to identify challenges and solutions but are rarely acknowledged in the scientific literature.

## Conclusions

This study investigated internal factors slowing down clinical trials, defined as those factors relating to clinical trials teams and sponsors only. In interviews with clinical trial staff working in research centres in SSA, we identified several such factors, which can be categorized broadly into the two themes “planning” and “site organisation”. We found that these internal factors are of high importance to trial staff, inhibit efficiency and may be addressed more easily as they are independent of external conditions. We argue that adequate and coherent planning, careful assessment of the context, performing dummy runs and clear task allocation may eliminate important internal factors that tend to slow down clinical trials. In the long run, strengthening management capacities may lead to improved portfolios, balanced workloads, reliable staffing and increased career options for trialists.

## Supporting information

S1 Text Interview guide. (PDF)
