# Supplementary material for: "You can save time if…"—A qualitative study on internal factors slowing down clinical trials in Sub-Saharan Africa
Source: PLoS One. 2017 Mar 16;12(3):e0173796. doi: 10.1371/journal.pone.0173796 (PMC5354381; doi:10.1371/journal.pone.0173796)
Supplement: S1 Text — (PDF) [file pone.0173796.s001.pdf]

Supporting information 1: Interview guide

Demographic background

- What kind of positions have you already had within clinical research?
- What was tested in your trials: drugs, vaccines, diagnostics or medical devices?
- In which phases of trials have you been involved in?
- For how many years have you been working in clinical trials?

Questions

- What is your opinion what is important for having a good quality in clinical trials? We defined quality as patient `s safety and rights and data integrity.
- Do you think that quality could be improved in clinical trials for improving patient`s safety and rights and data integrity? If yes: how?
- Is time being lost in the conduct of clinical trials for example with unnecessary repetition or by spending a lot of time for a small detail? If yes where?
- If I ask the other way around, do you think it would be possible to save time in the conduct of clinical trials? If yes where?
- Do you face any challenges in the trials?
- What might be future challenges in the trials?

This interview guide only focuses on the data presented in this manuscript.
